# Publisher Correction: Multistep nucleation of anisotropic molecules

**DOI:** 10.1038/s41467-021-25919-3

**Published:** 2021-09-16

**Authors:** Kazuaki Z. Takahashi, Takeshi Aoyagi, Jun-ichi Fukuda

**Affiliations:** 1 Research Center for Computational Design of Advanced Functional Materials, National Institute of Advanced Industrial Science and Technology (AIST), Tsukuba, Ibaraki Japan; 2 Department of Physics, Faculty of Science, Kyushu University, Fukuoka, Fukuoka Japan

**Keywords:** Molecular dynamics, Phase transitions and critical phenomena, Liquid crystals, Chemical physics

Correction to: *Nature Communications* 10.1038/s41467-021-25586-4, published online 06 September 2021

The original version of this Article contained errors in the subsection “Real-space density profiles of metastable clusters and critical nuclei” of the “Results” section. The hyperlinks to Fig. 3—Fig. 3, Fig. 3a, Fig. 3b, Fig. 3c—are mistakenly hyperlinked to Fig. 2.

This has been corrected in both the PDF and HTML versions of the Article.

The original version of this Article contained errors in the subsection “Dynamics of metastable clusters, critical nuclei and supercritical nuclei in the transition” of the “Results” section. The hyperlinks to Fig. 4—Fig. 4a, Fig. 4b, Fig. 4c—are mistakenly hyperlinked to Fig. 3.

This has been corrected in both the PDF and HTML versions of the Article.

