# Supplementary material for: Multistep nucleation of anisotropic molecules
Source: Nat Commun. 2021 Sep 6;12:5278. doi: 10.1038/s41467-021-25586-4 (PMC8421422; doi:10.1038/s41467-021-25586-4)
Supplement: Supplementary file 1 — Supplementary Information [file 41467_2021_25586_MOESM1_ESM.pdf]

Supplementary Information for

Multistep nucleation of anisotropic molecules

Kazuaki Z. Takahashi,[1*] Takeshi Aoyagi,[1] Jun-ichi Fukuda[2]

[1] Research Center for Computational Design of Advanced Functional Materials, National Institute of Advanced Industrial Science and Technology (AIST), Central 2, 1-1-1 Umezono, Tsukuba, Ibaraki 305-8568, Japan

[2] Department of Physics, Faculty of Science, Kyushu University, 744 Motooka, Nishi-ku, Fukuoka, Fukuoka 819-0395, Japan

*To whom correspondence should be addressed; E-mail: kazu.takahashi@aist.go.jp

Supplementary Figures

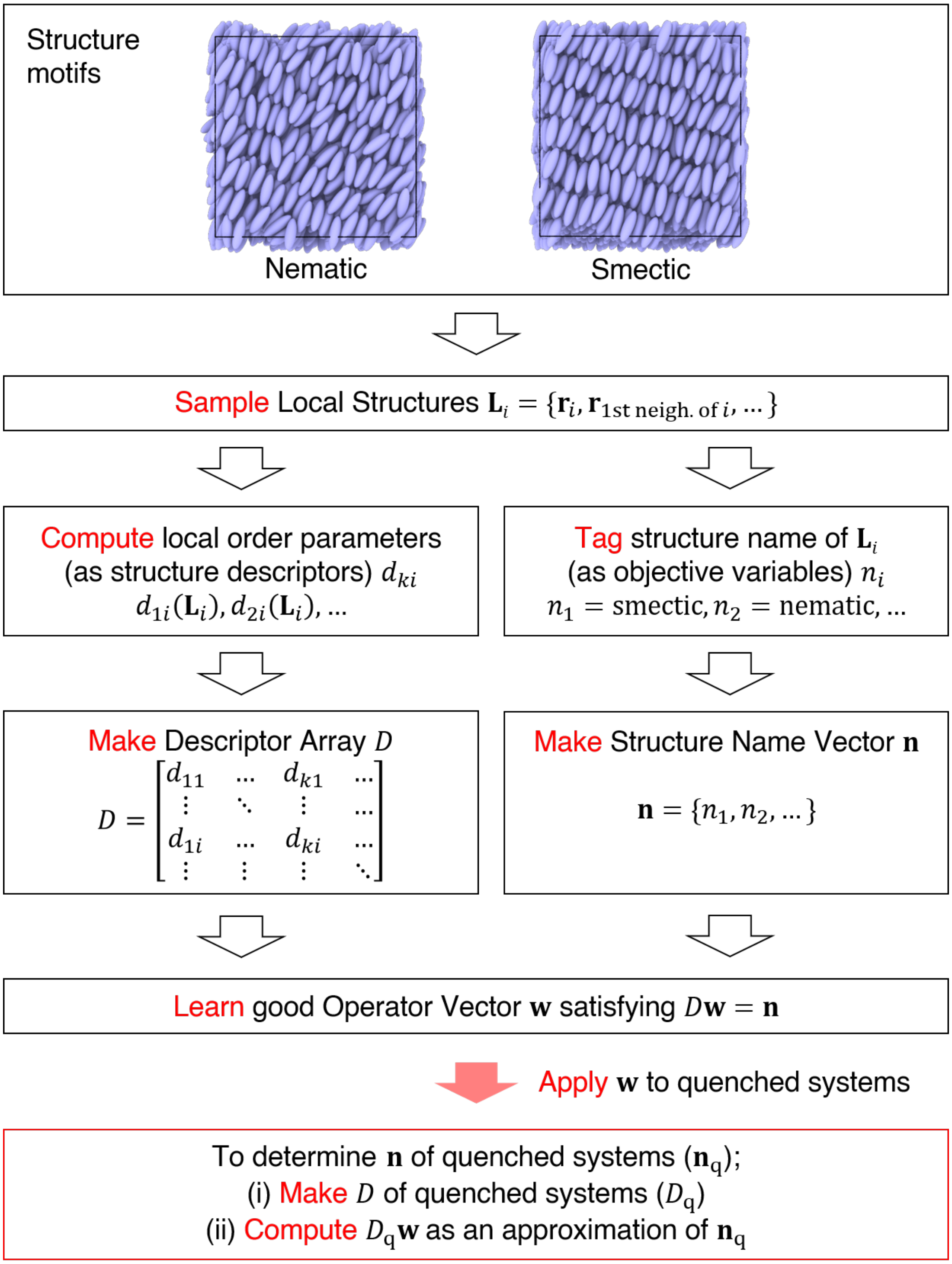

Supplementary Figure 1: Machine-learning flow using the Machine-Learning-aided Local Structure Analyzer specialized for this work.

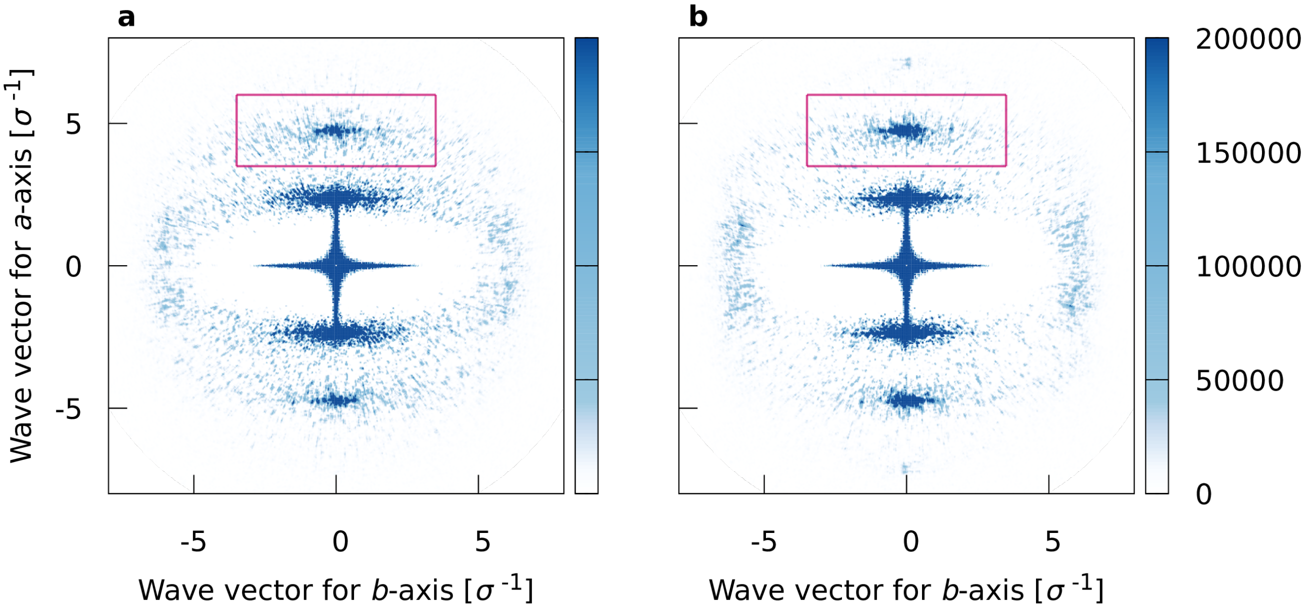

Supplementary Figure 2: Scattered X-ray patterns of 1 million SCGB particles at **a** $t = 0$, and **b** $t = 1.5\tau$. The patterns for fully nematic ($t = 0$) and partially smectic ($t = 1.5\tau$) coordinates were obviously different. Furthermore, corresponding to previous reports,[1,2] the difference in intensity between the two phases was considerable around the second harmonic (red-bounded areas). Therefore, the intensity was integrated over this area, and the integrated value was recorded along with the time evolution (results are shown in Fig. 1b of the main manuscript). Note that the X-ray scattering pattern is solely computed from quenched coordinates themselves; no results from the molecular cluster analysis are used.

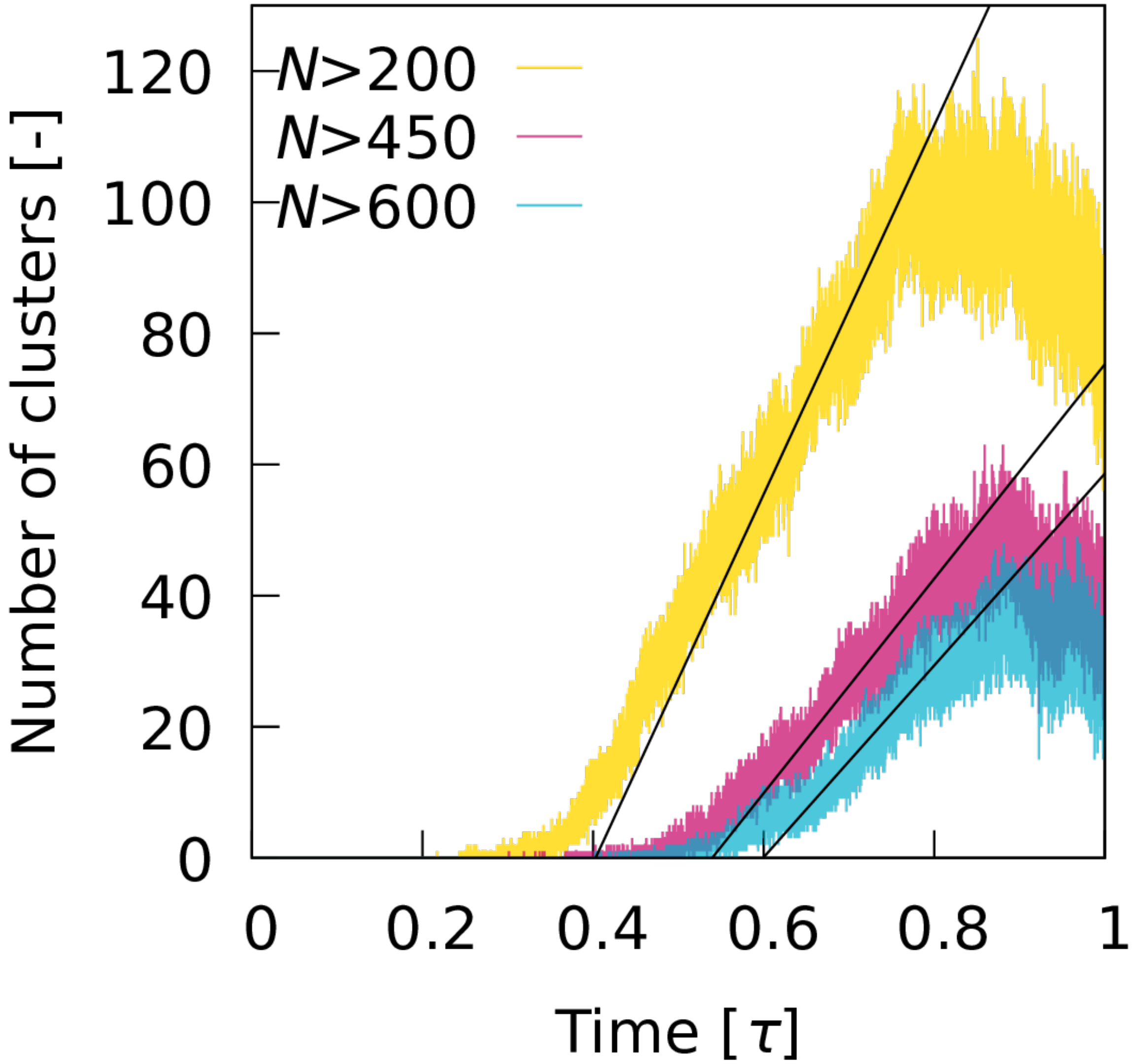

Supplementary Figure 3: Some examples of the time evolution for the number of supercritical nuclei assumed by threshold method (data were from the results of SCGB). A black line denotes a constant slope. The nucleation rate is estimated from the number of supercritical nuclei generated per unit time, per unit volume. To obtain the precise nucleation rate, the time interval in which the increment of supercritical nuclei is almost constant should be observed on systems with a large number of surrounding molecules not included to clusters. Our large systems of (SC)GB particles are sufficient to estimate the nucleation rate precisely. However, the calculation of nucleation rate is basically impossible without information concerning critical nuclei. The threshold method is a straightforward technique fixing the size of the critical nuclei to a certain value and then computing the nucleation rate. The computed nucleation rate is expected to converge with increasing fixed size. In this method, the converged value can be regarded as the true nucleation rate, and the minimum fixed size having the true nucleation rate can be regarded as the true size of critical nuclei. The fixed size is often called the threshold size.

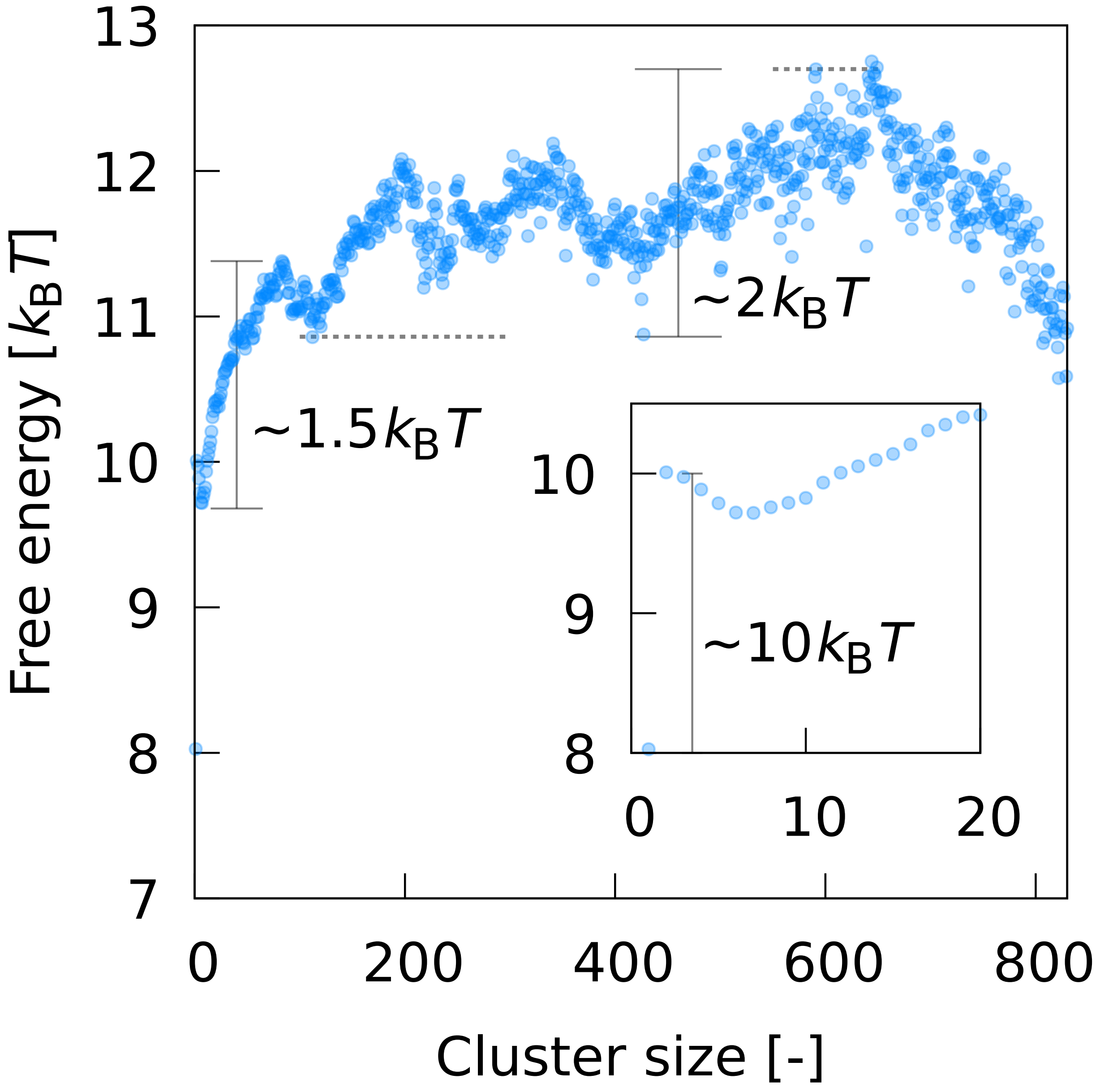

Supplementary Figure 4: Free energy minimum for each cluster size calculated for a GB system. Two metastable clusters around $N = 7$ and $N = 120$ are observed, corresponding to the MC1 and MC2 of SCGB, respectively. This fact indicates that at least MC1 and MC2 have universality beyond the difference in molecular models and initial structures. For the GB system, metastable clusters larger than MC2 are observed around N=230 and $N = 420$, and the critical nuclei are around $N = 650$. The energy barrier from MC1 to MC2 is $\sim 1.5k_\mathrm{B}T$, and the barrier from MC2 to CN is $\sim 2k_\mathrm{B}T$. The total energy barrier of a major pathway is $\sim 12.7k_\mathrm{B}T$ from the reference state (*i.e.*, nematic phase), corresponding to a weak first-order phase transition. The lower free energy barrier for SCGB than for GB is consistent with the design concept for SCGB.[3] Smaller energy barriers make it possible to sample a larger number of clusters, which yield more reliable statistics for nucleation phenomena. Therefore, SCGB systems were mainly investigated in this work.

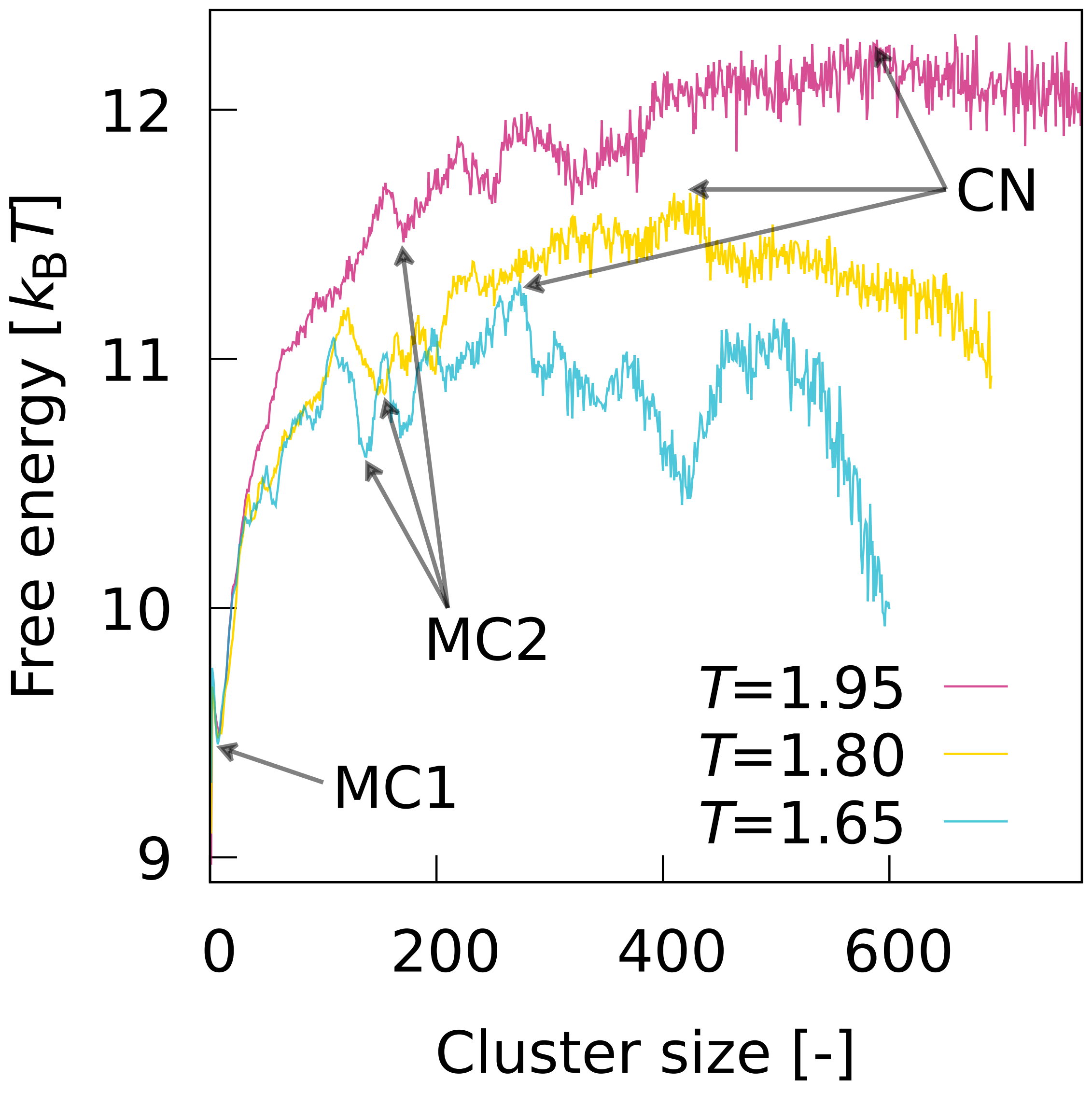

Supplementary Figure 5: Comparison of the free energy minima curves at $T = 1.95$ ($\Delta T = 0.30$), $T = 1.80$ ($\Delta T = 0.45$), and $T = 1.65$ ($\Delta T = 0.60$).

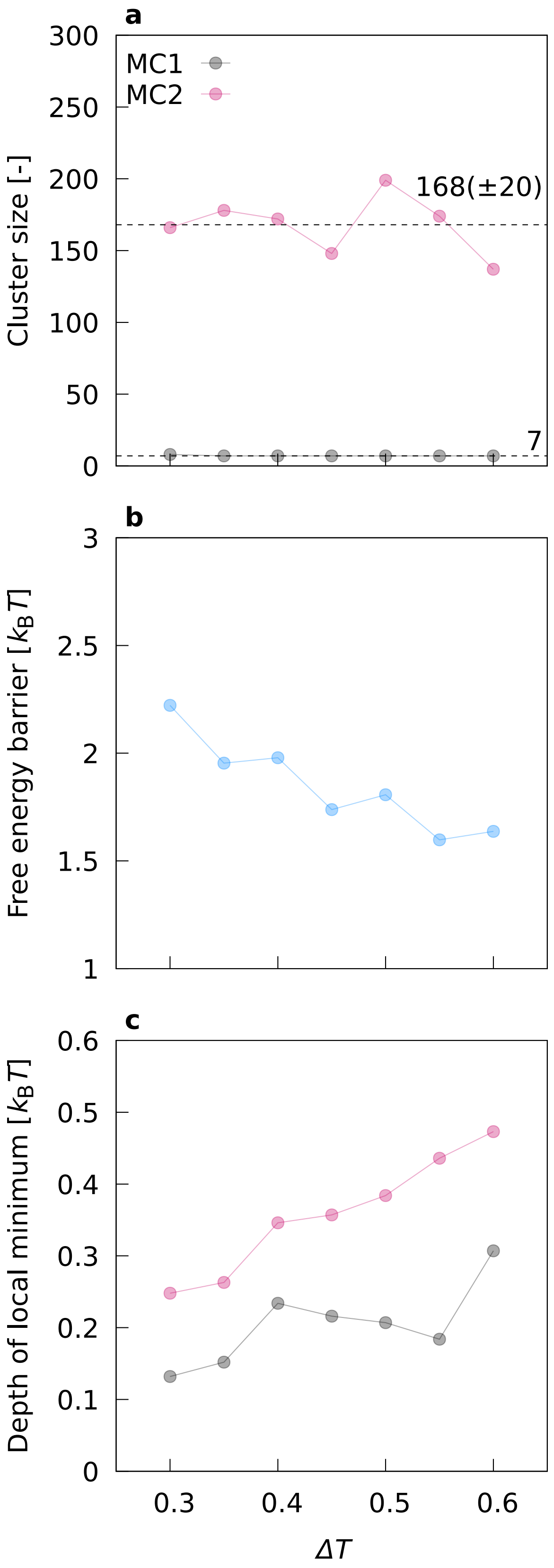

Supplementary Figure 6: **a** ΔT dependence of the cluster size of MC1 and MC2. **b** ΔT dependence of the height of the energy barriers from MC1 to MC2. **c** ΔT dependence of the depth of the local minimum from the top of the barrier for MC1 and MC2.

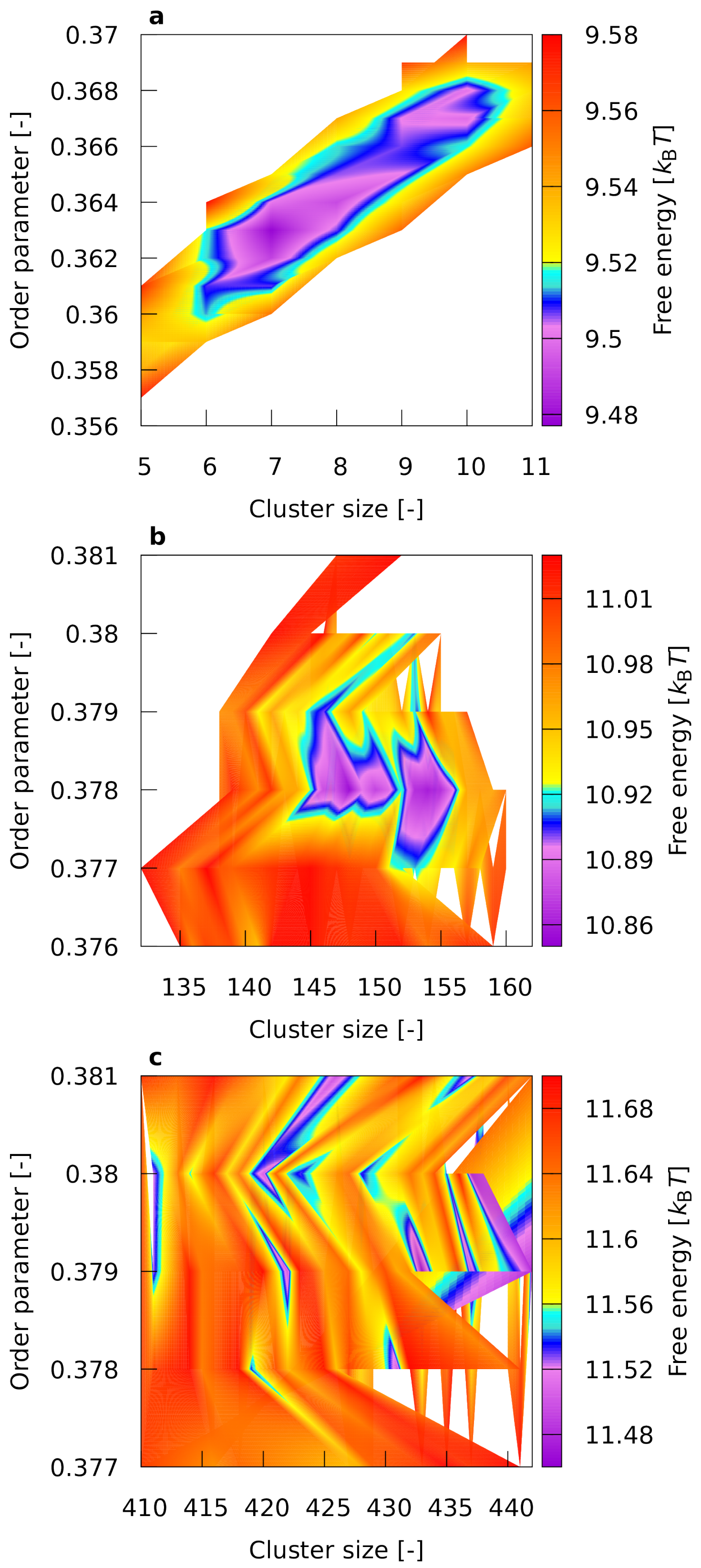

Supplementary Figure 7: Free energy contour map of all the states of **a** MC1, **b** MC2, and **c** CN, selected by the identification criteria: for MC1, $5 \leq N \leq 11$ and $0.357 \leq Q \leq 0.370$; for MC2, $132 \leq N \leq 162$ and $0.376 \leq Q \leq 0.381$; and for CN, $410 \leq N \leq 442$ and $0.377 \leq Q \leq 0.381$.

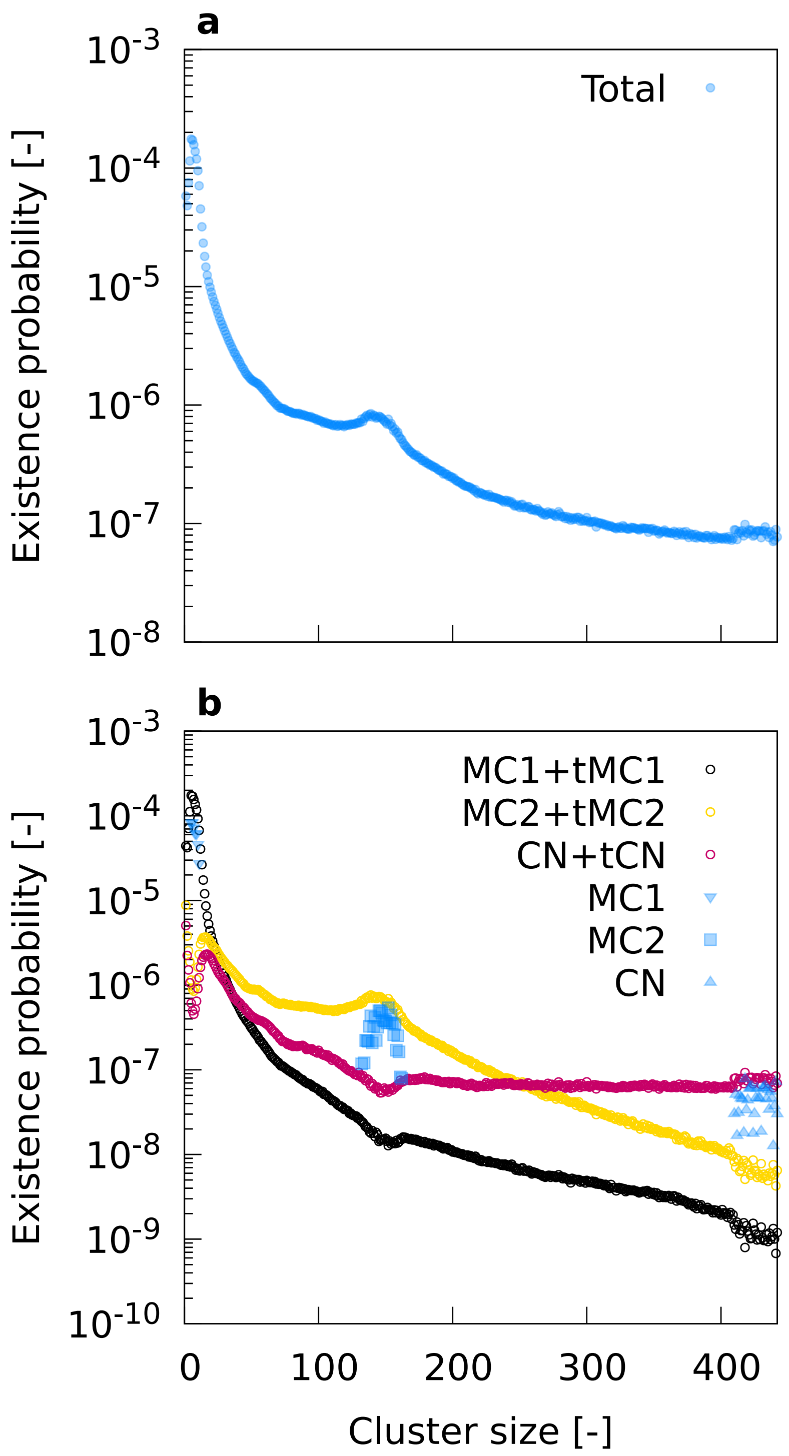

Supplementary Figure 8: Size distribution of the existence probabilities for **a** MC1, MC2, CN, tMC1, tMC2, and tCN clusters, and **b** MC1, MC2, and CN series. The distribution of the characteristic size for all clusters corresponds to the free energy landscape (cf. Fig. 1e of the main manuscript). The distributions for MC1, MC2, and the CN series are well captured by the range in characteristic size without any extreme outliers. Note that the data for Supplementary Fig. 8 were from cluster samples for which $0.80\tau \leq t \leq 0.90\tau$ corresponding to the free energy analysis.

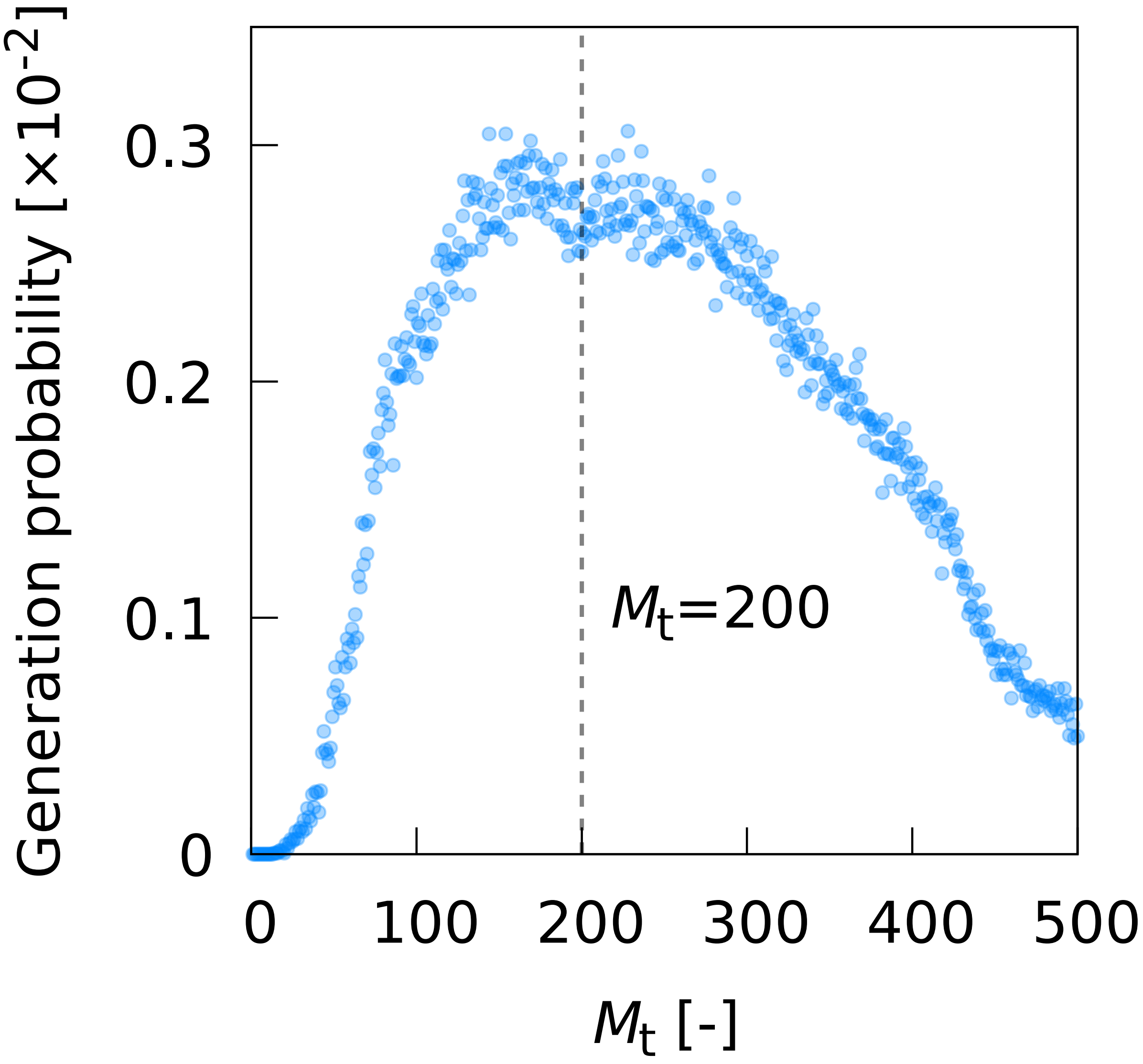

Supplementary Figure 9: Generation probability of newly born ScNs against the sum of the number of characteristic molecules included in the ScNs, M_t. The newly born ScNs during nucleation were distinguished and counted in the M_t values. The generation probability was calculated simply from the ratio of the count number to the total number of ScNs. The distribution features a dip at $M_\mathrm{t} = 200$, indicating a bipolarization in the generation probability for ScNs. Therefore, we classified the ScNs into two groups using the threshold $M_\mathrm{t} = 200$. Note that the data for Supplementary Fig. 9 were from cluster samples for which $0.50\tau \leq t \leq 0.80\tau$ corresponding to nucleation.

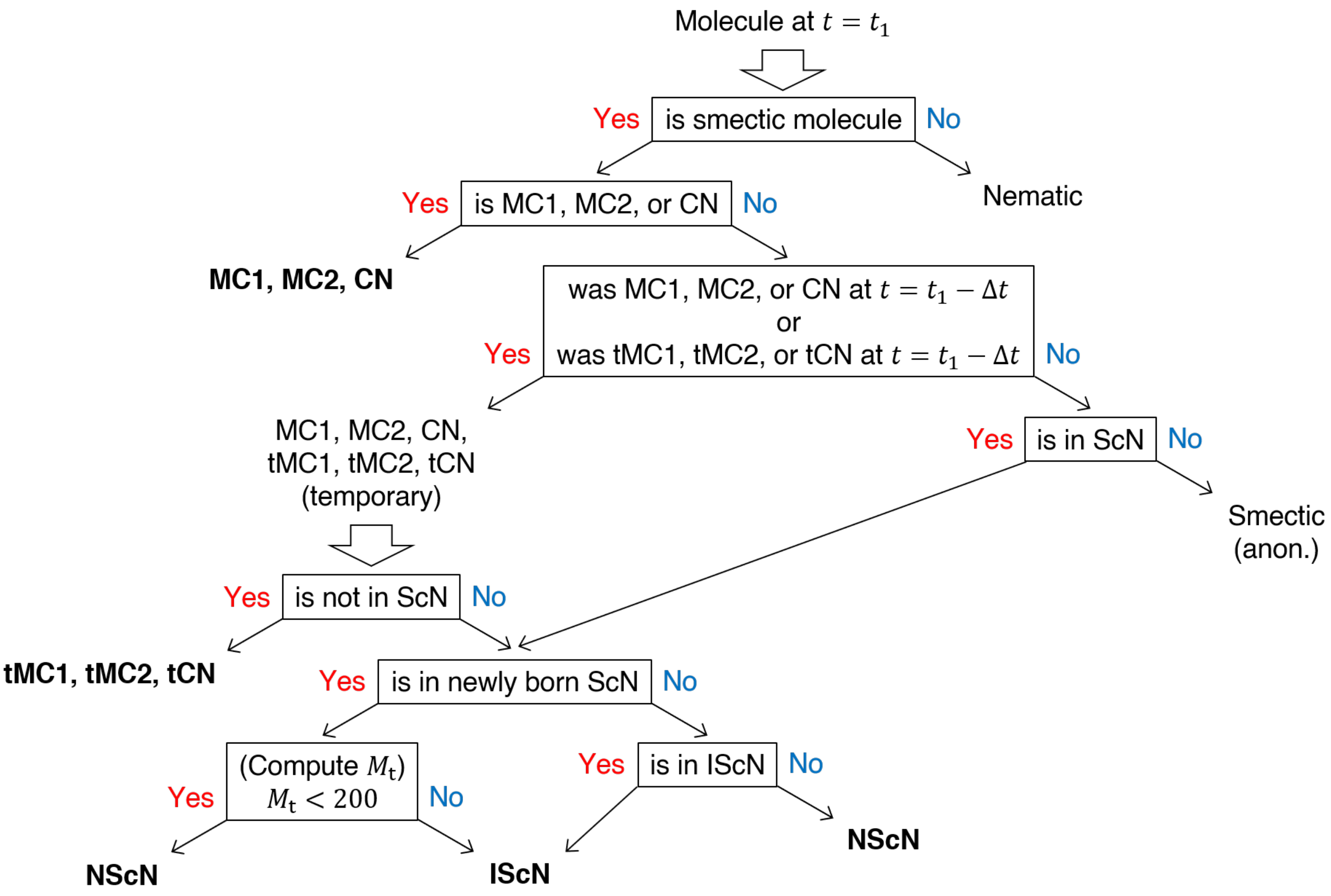

Supplementary Figure 10: Flow to assign the molecules to characteristic clusters, nematic phase, IScN, or NScN.

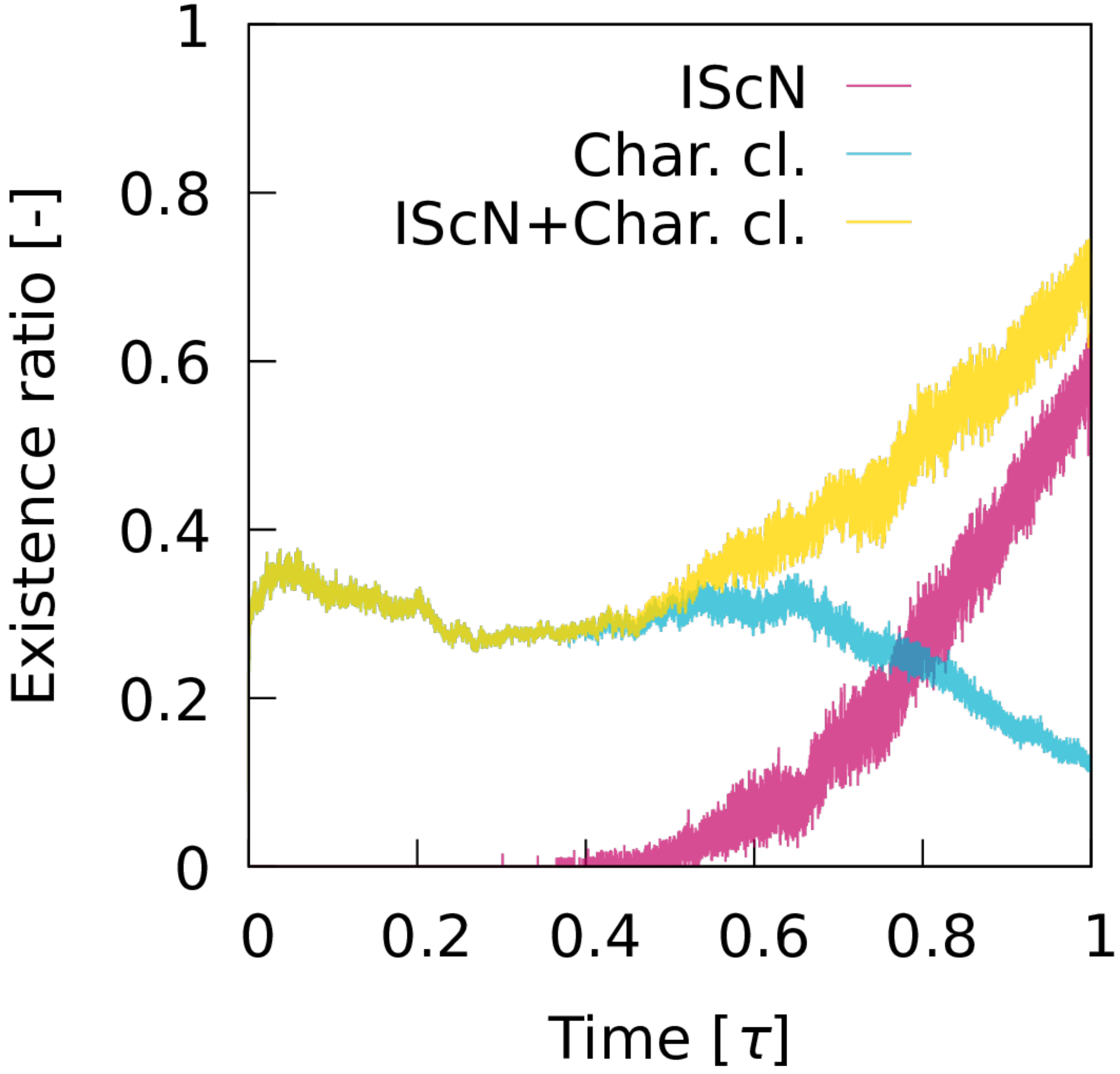

Supplementary Figure 11: Time evolution for the existence ratio of molecules belonging to IScNs or characteristic clusters in comparison with the total smectic molecules. The sum of the ratios (IScN + Char. cl.) is also plotted, which monitors the number of molecules on or through the major pathway (denoted hereafter by "major pathway molecules"). The ratio for characteristic clusters was kept at ~ 0.3 with $t < 0.64\tau$, but decreased along with a strong increase in the ratio for IScNs at $t > 0.64\tau$, implying that characteristic clusters were spent in forming IScNs. At $t \sim 0.80\tau$ the number of major pathway molecules exceeded half the total number of smectic molecules in the system, and the IScN molecules were the largest share in the major pathway molecules. The ratio for IScNs steadily increased even after that. Obviously, the molecules associated with the major pathway, especially the IScNs, affect the results obtained from the X-ray scattering pattern.

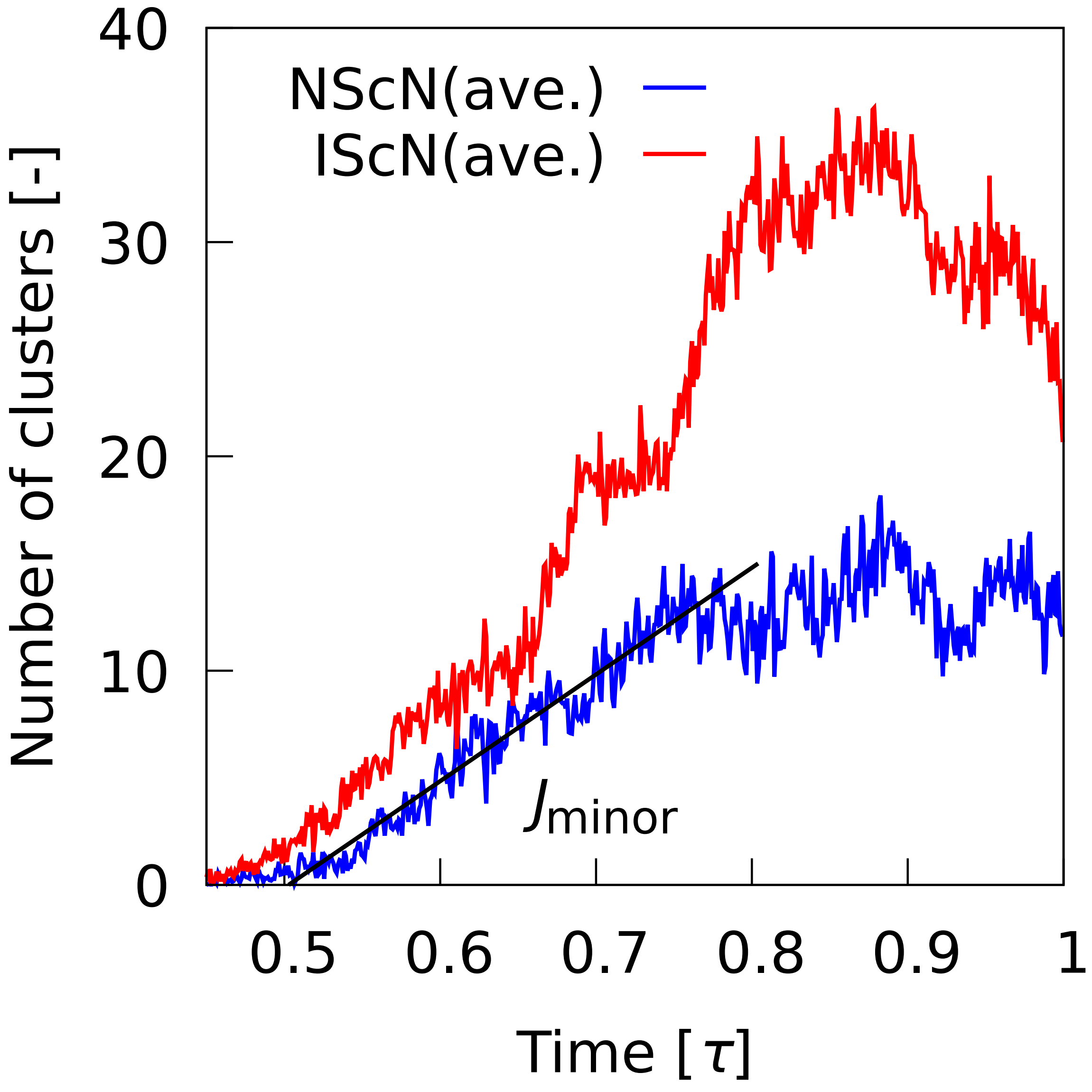

Supplementary Figure 12: Time evolution for the number of NScNs averaged per 0.001τ. The time evolution for the number of IScNs averaged per 0.001τ is also plotted. NScNs are steadily increasing during $0.50\tau < t < 0.75\tau$, and then saturate. This reflects the dynamics of clusters on the minor pathway, *i.e.*, the local minima of free energy trapping the relatively stable clusters has little effect on the unstable process. Also, the saturation at $t > 0.75\tau$ clearly indicates that NScNs contribute little to the increase in the smectic molecules in the systems, and therefore in increasing the intensity of X-ray scattering. The solid black line is the fitted slope for NScN at $0.50\tau < t < 0.75\tau$, indicating the magnitude of nucleation for the minor pathway. The slope is close to that of 1st nucleation process of IScN. In fact, the nucleation rate of NScN, J_{minor}, computed from the slope, is $1.49 \times 10^{-5}\sigma^{-3}\tau^{-1}$.

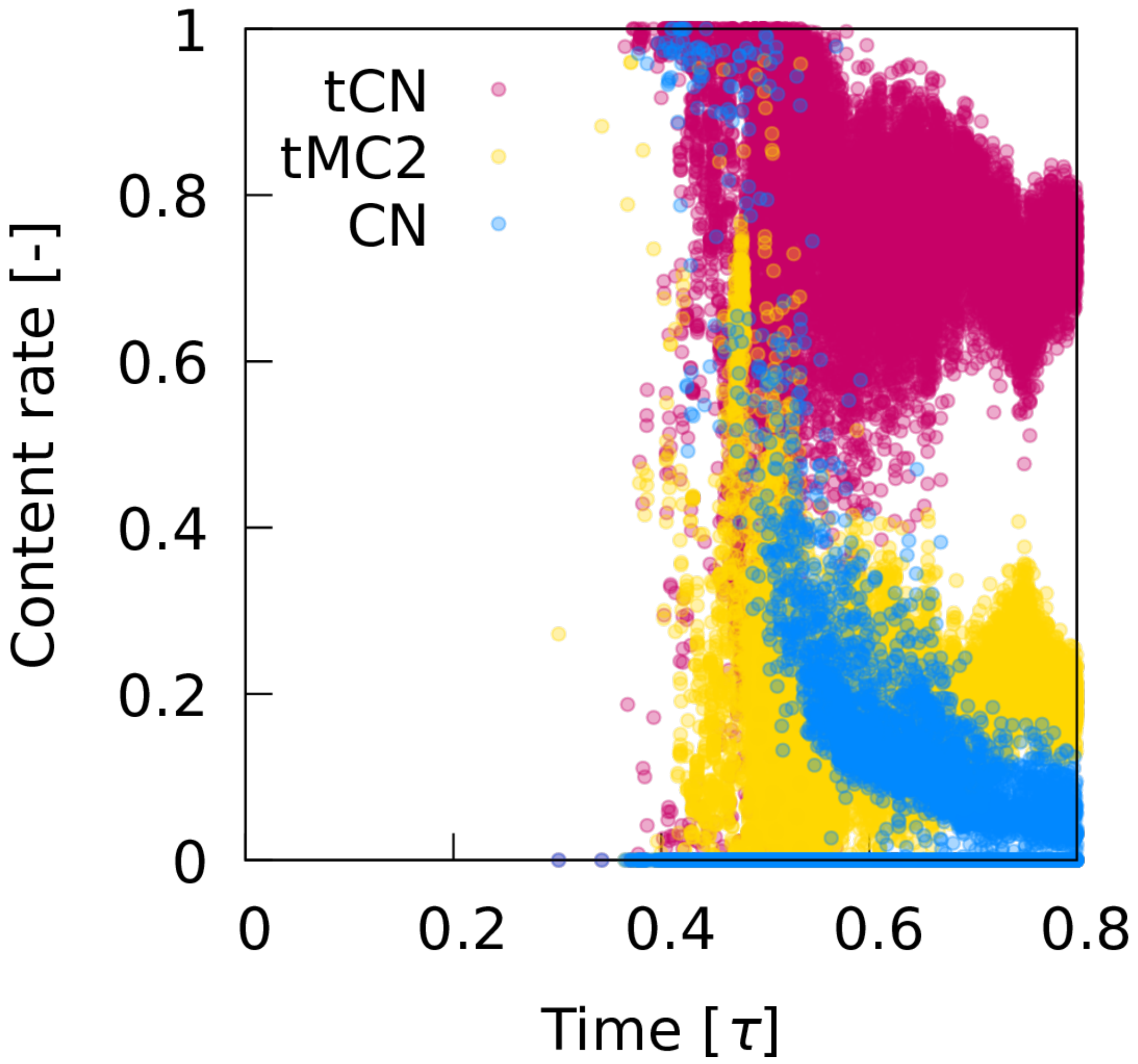

Supplementary Figure 13: Time evolution for the content rate of tCN, tMC2, and CN molecules included in newly born IScNs. The definition of content rate is simple; the number of specific species of characteristic molecules per the number of total characteristic molecules included in IScNs. Overall, tCN is the largest component, indicating that the IScNs are directly induced by tCNs. The contributions of tMC2 and CN are relatively high at $t < 0.55\tau$, and then diminish. The contribution of tMC2 is higher than that of CN. Note that tCN, tMC2, and CN are almost all the characteristic molecules included in IScNs.

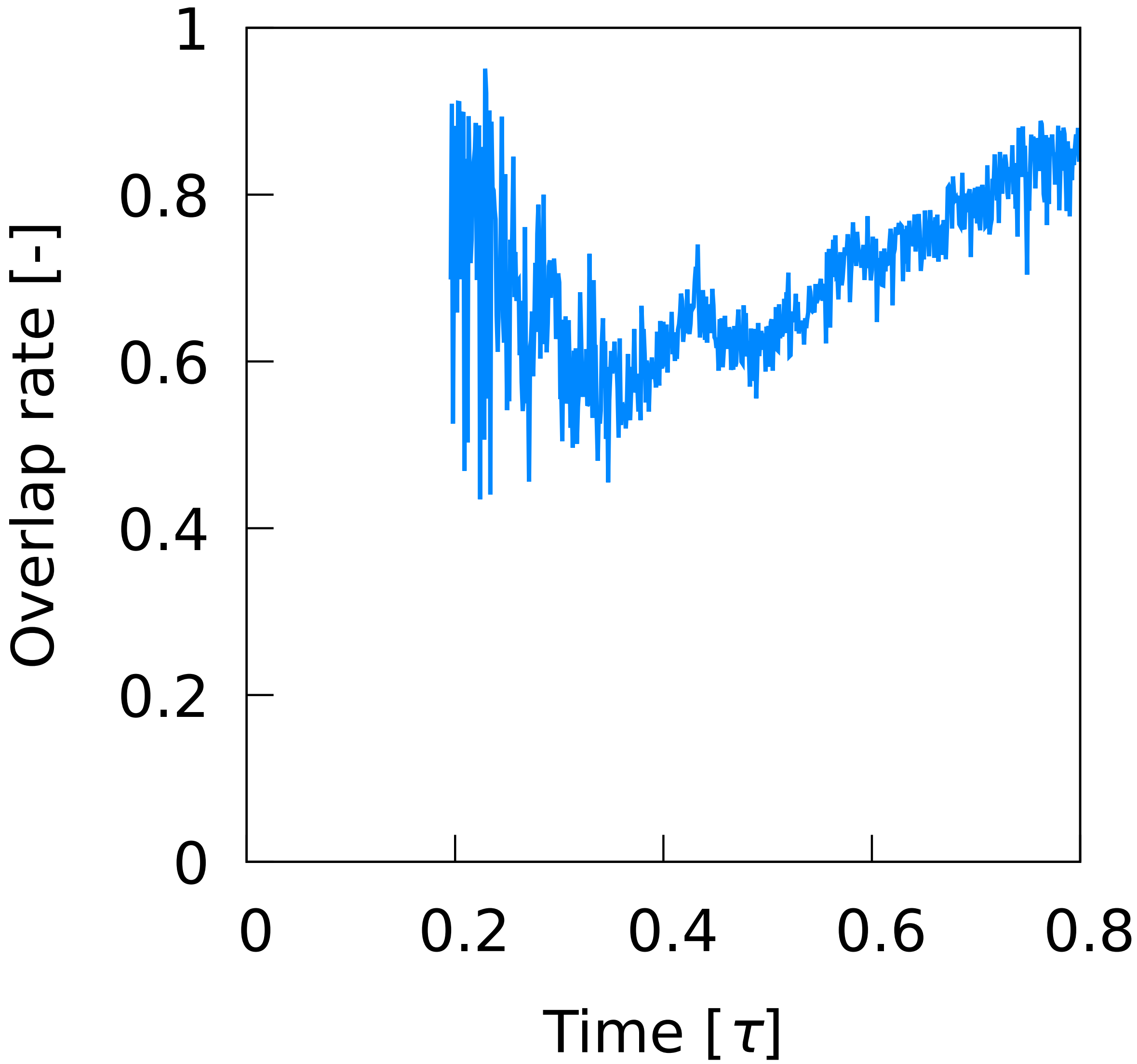

Supplementary Figure 14: Time evolution of overlap rate computed from the neighbor analysis between tMC2 molecules and the region occupied by tMC2, tCN, and IScN molecules of the end of 3rd nucleation ($t = 0.80\tau$). The neighboring rule is consistent to ML scheme and molecular cluster analysis. The definition of overlap rate is simple; the number of overlapped tMC2 molecules per the number of total tMC2 in system. The overlap rate is consistently the high of ~ 0.6 at $t < 0.50\tau$. Furthermore, the rate steadily increases at $t > 0.50\tau$, indicating that the molecular assembly of tMC2s becomes the backbone of IScN formation.

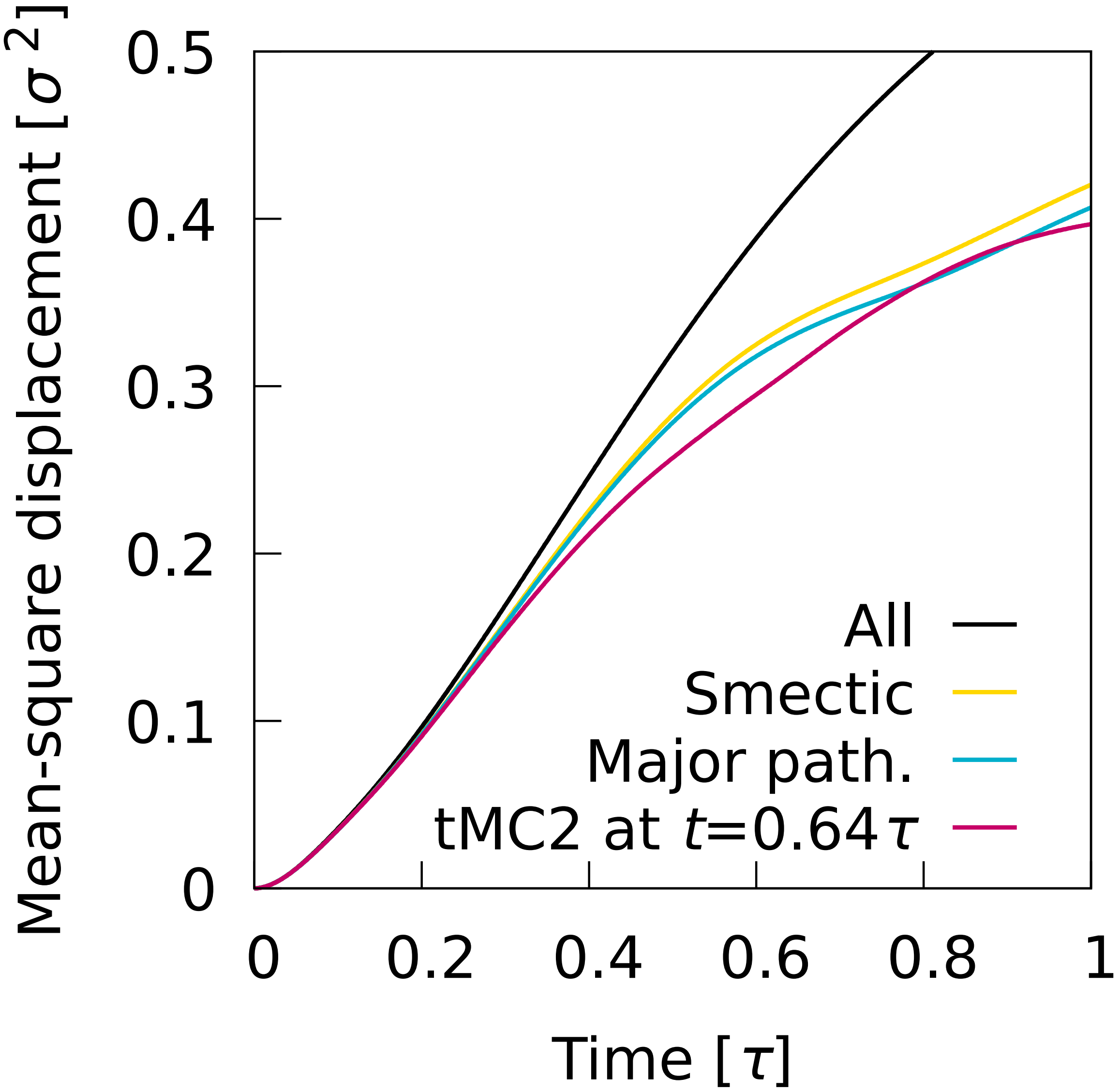

Supplementary Figure 15: Mean-square displacement (MSD) for all molecules, and molecules identified to be smectic or major pathway molecules at the end of 3rd nucleation ($t = 0.80\tau$). MSD for molecules identified to be tMC2 at the beginning of 2nd nucleation ($t = 0.64\tau$) is also plotted. The mobility of each group is consistent to energy stability. At $0.40\tau < t < 0.64\tau$, the mobility of tMC2 molecules is clearly low comparing that of major pathway molecules. At $t > 0.64\tau$, the mobility of tMC2 gradually increases, and coincides to that of major pathway molecules at the time from the end of 3rd nucleation to the end of 3rd plateau.

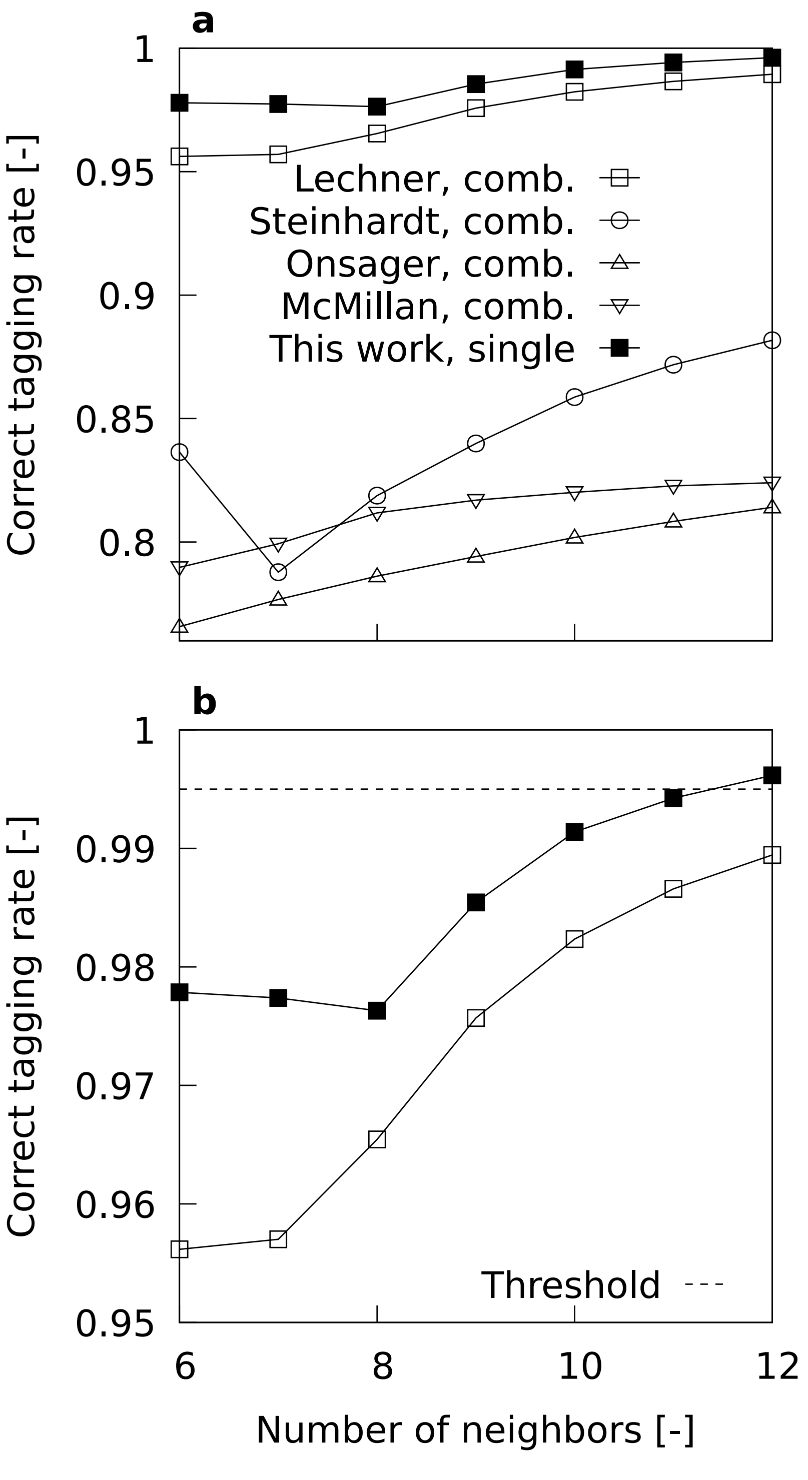

Supplementary Figure 16: Comparison of accuracies of local order parameters for classification of nematic or smectic structures. **a** Plots the local order parameter developed in this work and conventional ones.[4] **b** Plots the results near the threshold for classification accuracy. Note that the results of conventional ones are from combination of two order parameters for redeeming poor accuracy. Our developed single order parameter outstrips the combinations of conventional ones. For precise description of nucleation process, the classification accuracy over $C = 0.995$ is required.[4] Considering the first to twelfth neighbors satisfies the criterion. Therefore, $Q_{l=2}(i)$ was used for this work.

Supplementary References

1. Bates, M. A. & Luckhurst, G. R. X-ray scattering patterns of model liquid crystals from computer simulation: Calculation and analysis. *The Journal of Chemical Physics* **118**, 6605–6614 (2003).

2. Skačej, G. & Zannoni, C. Molecular simulations elucidate electric field actuation in swollen liquid crystal elastomers. *Proceedings of the National Academy of Sciences of the United States of America* **109**, 10193–10198 (2012).

3. Berardi, R., Zannoni, C., Lintuvuori, J. S. & Wilson, M. R. A soft-core Gay–Berne model for the simulation of liquid crystals by Hamiltonian replica exchange. *The Journal of Chemical Physics* **131**, 174107 (2009).

4. Doi, H., Takahashi, K. Z., Tagashira, K., Fukuda, J.-i. & Aoyagi, T. Machine learning-aided analysis for complex local structure of liquid crystal polymers. *Scientific Reports* **9**, 16370 (2019).
