# Supplementary material for: Multistep nucleation of anisotropic molecules
Source: Nat Commun. 2021 Sep 6;12:5278. doi: 10.1038/s41467-021-25586-4 (PMC8421422; doi:10.1038/s41467-021-25586-4)
Supplement: Supplementary file 3 — Description of Additional Supplementary Files [file 41467_2021_25586_MOESM3_ESM.pdf]

Description of Additional Supplementary Files

File Name: Supplementary Movie 1
Description: Visualization of the smectic nucleation and growth trajectory of the SCGB system. The tMC2, tCN, lScN, MC2, and CN molecules are shown in yellow, red, blue, white and violet, respectively. Molecules that belong to none of them are not depicted. Colour change of molecules means that the type of clusters the molecules belong to has been changed, and the disappearance means that the molecules no longer belong to any of the clusters.
